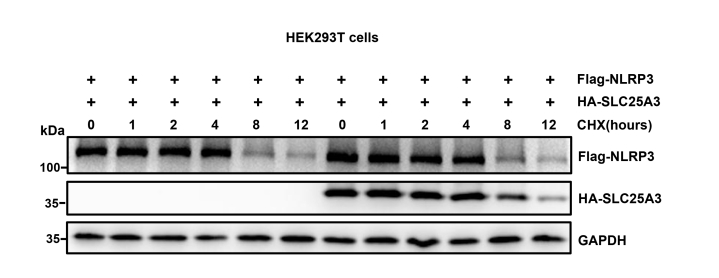# SLC25A3 negatively regulates NLRP3 inflammasome activation by restricting the function of NLRP3

**DOI:** 10.1016/j.jbc.2024.107233

**Published:** 2024-03-27

**Authors:** Feng Xiao, Yaling Jia, Simeng Zhang, Nanfang Liu, Xuelong Zhang, Tianci Wang, Jialu Qiao, Ge Yang, Xu Che, Keli Chen, Pan Pan, Lingli Zhou, Binlian Sun, Jun Chen, Pin Wan

**Affiliations:** 1 Hubei Key Laboratory of Cognitive and Affective Disorders, Institute of Biomedical Sciences, School of Medicine, Jianghan University, Wuhan, China; 2 Department of Urology, The Third Affiliated Hospital of Shenzhen University (Luohu Hospital Group), Shenzhen, China; 3 Key Laboratory of Viral Pathogenesis & Infection Prevention and Control (Jinan University), Ministry of Education, Guangzhou, China; 4 Foshan Institute of Medical Microbiology, Foshan, China; 5 State Key Laboratory of Virology, College of Life Sciences, Wuhan University, Wuhan, China; 6 State Key Laboratory of Bioactive Molecules and Druggability Assessment, Jinan University, Guangzhou, China

**Keywords:** innate immune response, NLRP3 inflammasome activation, SLC25A3, mitochondria, pyroptosis

## Abstract

The NACHT, leucine-rich repeat, and pyrin domains-containing protein 3 (collectively known as NLRP3) inflammasome activation plays a critical role in innate immune and pathogenic microorganism infections. However, excessive activation of NLRP3 inflammasome will lead to cellular inflammation and tissue damage, and naturally it must be precisely controlled in the host. Here, we discovered that solute carrier family 25 member 3 (SLC25A3), a mitochondrial phosphate carrier protein, plays an important role in negatively regulating NLRP3 inflammasome activation. We found that SLC25A3 could interact with NLRP3, overexpression of SLC25A3 and knockdown of SLC25A3 could regulate NLRP3 inflammasome activation, and the interaction of NLRP3 and SLC25A3 is significantly boosted in the mitochondria when the NLRP3 inflammasome is activated. Our detailed investigation demonstrated that the interaction between NLRP3 and SLC25A3 disrupted the interaction of NLRP3-NEK7, promoted ubiquitination of NLRP3, and negatively regulated NLRP3 inflammasome activation. Thus, these findings uncovered a new regulatory mechanism of NLRP3 inflammasome activation, which provides a new perspective for the therapy of NLRP3 inflammasome-associated inflammatory diseases.

NOD-like receptors (NLRs), belonging to the pattern recognition receptors family members, play critical roles in innate immunity and pathogen infections (1, 2, 3). NACHT, leucine-rich repeat, and pyrin domain-containing protein 3 (NLRP3) is the most extensively concerned and studied receptor in NLRs (4, 5). NLRP3 inflammasome, a multiprotein complex, is mainly composed of NLRP3, apoptosis-associated speck-like protein containing a CARD (ASC), and caspase-1 (6, 7). It is capable of being activated by pathogen-associated molecular patterns or damage-associated molecular patterns, including virus, fungi, ATP, monosodium urate (MSU), silica crystals, fibrillar amyloid-β peptide, and aluminum salt crystals (8, 9, 10, 11, 12, 13). NLRP3 inflammasome activation regulates maturation and secretion of pro-inflammatory cytokines (such as interleukin 1β [IL-1β]), and as well as cleavage of gasdermin D (GSDMD), which promotes a lytic form of cell death called pyroptosis (14, 15). IL-1β can initiate multiple signaling pathways to drive inflammatory response, and N-terminal domain of GSDMD (GSDMD cleaved by active caspase-1) forms GSDMD pores in the plasma membrane to trigger pyroptosis (14). NLRP3 inflammasome activation plays a critical role in host immunity (6, 15). However, excessive activation of NLRP3 inflammasome will lead to tissue damage and immune disease (2, 16). Although the mechanisms of NLRP3 inflammasome activation are very clear, negative regulations of NLRP3 inflammasome activation remain largely unknown.

It has also been reported that never in mitosis A (NIMA)-related kinase-7 (NEK7) is essential for NLRP3 inflammasome activation and NEK7 can directly bind to NLRP3 when NLRP3 inflammasome is activated (17, 18, 19). Influenza A viruses limited NLRP3-NEK7-complex formation and NLRP3 inflammasome activation in human macrophages (17). Deubiquitination of PLK4 facilitates its binding to and phosphorylation of NEK7 at Ser204. NEK7 phosphorylation in turn limits the interaction of NLRP3-NEK7, which is required for NLRP3 inflammasome activation (20). Ubiquitination is a critical modification for the function of NLRP3. Ubiquitination of NLRP3 could frequently suppress the activation of NLRP3 inflammasome (21, 22, 23, 24, 25, 26). For example: E3 ligase enzymes tripartite motif (TRIM)31 and MARCH7 could ubiquitinate NLRP3 and lead to its degradation, which negatively regulates the activation of NLRP3 inflammasome (21, 27); E3 ligase enzymes, TRIM24 and TRIM65, could ubiquitinate NLRP3 to negatively regulate the activation of NLRP3 inflammasome (28, 29).

Solute carrier family 25 member 3 (SLC25A3), the inner mitochondrial membrane phosphate transporter, provides inorganic phosphate to the mitochondrial matrix (30, 31). It has been uncovered that decrease of SLC25A3 protein will lead to diminished mitochondrial ATP synthesis rates (30). Therefore, function of SLC25A3 is essential for ATP production in mitochondria. Interestingly, mammalian phosphate carrier protein SLC25A3 can also transport copper both invitro and *in vivo* (32). Recently, it has been showed that SLC25A3 deletion could induce mitochondrial energy dysfunction drives remodeling of the cardiac mitochondrial protein acylome (33). However, the role of SLC25A3 in regulation of NLRP3 inflammasome activation has not been uncovered.

In this study, we elucidate the mechanism underling suppression of NLRP3 inflammasome activation. The results revealed that SLC25A3 could interact with NLRP3, and overexpression of SLC25A3 negatively regulated NLRP3 inflammasome activation in THP-1-derived macrophages, and knockdown of SLC25A3 could accelerate NLRP3 inflammasome activation in THP-1 derived macrophages and bone marrow-derived macrophages (BMDMs). Moreover, the interaction of NLRP3 and SLC25A3 was significantly enhanced in the mitochondria when NLRP3 inflammasome was activated. The interaction of NLRP3 and SLC25A3 disrupted the interaction of NLRP3-NEK7, promoted ubiquitination of NLRP3, and finally negatively regulated NLRP3 inflammasome activation. Therefore, the work uncovered a distinct mechanism by which SLC25A3 negatively regulates NLRP3 inflammasome activation.

## Results

### SLC25A3 interacts with NLRP3

**Figure 1:**
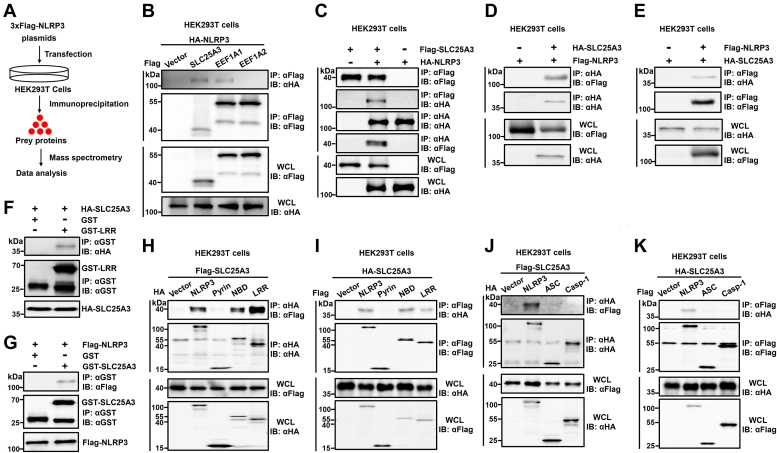
**SLC25A3 interacts with NLRP3.***A*, the schematic of mass spectrometry. *B*, HA-NLRP3 was cotransfected with Vector-Flag, Flag-SLC25A3, Flag-EEF1A1, or Flag-EEF1A2 into the HEK293T cells. The cell lysates were immunoprecipitated with anti-Flag antibody, and then immunoblotted with indicated antibodies. *C*, HEK293T cells were transfected with Flag-SLC25A3 and Vector-HA, Flag-SLC25A3 and HA-NLRP3, or Vector-Flag and HA-NLRP3, respectively. The cell lysates were immunoprecipitated with anti-Flag or anti-HA antibodies and then immunoblotted with indicated antibodies. *D*, HEK293T cells were transfected with Flag-NLRP3 and Vector-HA, or HA-SLC25A3 and Flag-NLRP3 as indicated in the figure, respectively. The cell lysates were immunoprecipitated with anti-HA antibodies and then immunoblotted with indicated antibodies. *E*, HEK293T cells were transfected with HA-SLC25A3 and Vector-Flag, or HA-SLC25A3 and Flag-NLRP3 as indicated in the figure, respectively. The cell lysates were immunoprecipitated with anti-Flag antibodies and then immunoblotted with indicated antibodies. *F*, HEK293T were transfected with HA-SLC25A3. The cell lysates were mixed with GST or GST-LRR, then immunoprecipitated with anti-GST antibodies and finally immunoblotted with indicated antibodies. *G*, HEK293T cells were transfected with Flag-NLRP3. The cell lysates were mixed with GST or GST-LRR, then immunoprecipitated with anti-GST antibodies and finally immunoblotted with indicated antibodies. *H*, HEK293T cells were transfected with Flag-SLC25A3 and Vector-HA, HA-NLRP3, HA-Pyrin, HA-NBD, or HA-LRR as indicated in the figure. The cell lysates were immunoprecipitated with anti-HA antibodies and then immunoblotted with indicated antibodies. *I*, HEK293T cells were transfected with HA-SLC25A3 and Vector-Flag, Flag-NLRP3, Flag-Pyrin, Flag-NBD, or Flag-LRR as indicated in the figure. The cell lysates were immunoprecipitated with anti-Flag antibodies and then immunoblotted with indicated antibodies. *J*, HEK293T cells were transfected with Flag-SLC25A3 and Vector-HA, HA-NLRP3, HA-ASC, or HA-caspase-1 as indicated in the figure. The cell lysates were immunoprecipitated with anti-HA antibodies, and then immunoblotted with indicated antibodies. *K*, HEK293T cells were transfected with HA-SLC25A3 and Vector-Flag, Flag-NLRP3, Flag-ASC, or Flag-caspase-1 as indicated in the figure. The cell lysates were immunoprecipitated with anti-Flag antibodies and then immunoblotted with indicated antibodies. GST, glutathione-*S*-transferase; HA, hemeagglutinin; LRR, leucine-rich repeat; NBD, Nucleotide-binding domain; NLRP3, NACHT, leucine-rich repeat, and pyrin domain-containing protein 3; SLC25A3, solute carrier family 25 member 3.

In order to reveal the regulatory mechanism of NLRP3 inflammasome activation, we initially screened targeted proteins interacting with NLRP3 by mass spectrometry in HEK293T cells (Fig 1*A*). Three targeted proteins (SLC25A3, EEF1A1, and EEF1A2) were identified through mass spectrometry (Table 1). The result indicated that SLC25A3 and EEF1A1 could interact with NLRP3, and the interaction between NLRP3 and SLC25A3 is stronger (Fig. 1*B*). We found that targeted protein SLC25A3 might interact with NLRP3. To reveal if the interaction between NLRP3 and SLC25A3 is reliable, we had conducted different experiments. First, coimmunoprecipitation (Co-IP) confirmed that NLPR3 could interact with SLC25A3 in HEK293T cells (Fig. 1, *C*–*E*). Next, glutathione-*S*-transferase pull-down confirmed that NLRP3 or leucine-rich repeat domain of NLRP3 could interact with SLC25A3 *in vitro* (Fig. 1, *F* and *G*); finally, Co-IP confirmed that nucleotide-binding domain and leucine-rich repeat domains of NLRP3 could interact with SLC25A3 in HEK293T cells (Fig. 1, H and I). The NLRP3 inflammasome comprises NLRP3, ASC, and caspase-1 (15). To reveal the specificity of their interaction between NLRP3 and SLC25A3, we cotransfected SLC25A3 with NLRP3, ASC, or caspase-1 in HEK293T cells, respectively. The result indicated that SLC25A3 could specially interact with NLRP3 (Fig. 1, *J* and *K*). Overall, we demonstrated that SLC25A3 could interact with NLRP3.

**Table 1:** The targeted proteins interacting with NLRP3 by mass spectrometry in HEK293T cells

N	Unused	Total	%Cov	%Cov(50)	%Cov(95)	Accession	Name
1	37.15	37.15	25.0000	17.7600	15.2500	sp|Q96P20|NLRP3_HUMAN	NACHT, LRR and PYD domains-containing protein 3 OS=*Homo sapiens* GN=NLRP3 PE=1 SV=3
2	25.05	25.05	24.0100	18.8500	16.4900	sp|P38646|GRP75_HUMAN	Stress-70 protein, mitochondrial OS=*Homo sapiens* GN=HSPA9 PE=1 SV=2
3	16.69	16.69	22.3600	16.4600	15.3700	sp|P04264|K2C1_HUMAN	Keratin, type II cytoskeletal 1 OS=*Homo sapiens* GN=KRT1 PE=1 SV=6
4	6.73	6.73	19.5800	18.7800	13.3200	sp|P35527|K1C9_HUMAN	Keratin, type I cytoskeletal 9 OS=*Homo sapiens* GN=KRT9 PE=1 SV=3
5	6.27	6.27	17.0600	9.3900	7.5120	sp|P35908|K22E_HUMAN	Keratin, type II cytoskeletal 2 epidermal OS=*Homo sapiens* GN=KRT2 PE=1 SV=2
6	6.07	6.07	11.9900	6.1640	6.1640	sp|P13645|K1C10_HUMAN	Keratin, type I cytoskeletal 10 OS=*Homo sapiens* GN=KRT10 PE=1 SV=6
7	6.06	6.06	9.9070	4.4890	4.4890	sp|P11142|HSP7C_HUMAN	Heat shock cognate 71 kDa protein OS=*Homo sapiens* GN=HSPA8 PE=1 SV=1
7	0	6	22.8100	16.9600	16.9600	tr|E9PQQ4|E9PQQ4_HUMAN	Heat shock cognate 71 kDa protein (Fragment) OS=*Homo sapiens* GN=HSPA8 PE=1 SV=1
7	0	6	21.9100	16.2900	16.2900	tr|E9PQK7|E9PQK7_HUMAN	Heat shock cognate 71 kDa protein (Fragment) OS=*Homo sapiens* GN=HSPA8 PE=1 SV=1
7	0	6	20.8600	15.5100	15.5100	tr|E9PLF4|E9PLF4_HUMAN	Heat shock cognate 71 kDa protein (Fragment) OS=*Homo sapiens* GN=HSPA8 PE=1 SV=1
7	0	6	21.3100	15.8500	15.8500	tr|E9PK54|E9PK54_HUMAN	Heat shock cognate 71 kDa protein (Fragment) OS=*Homo sapiens* GN=HSPA8 PE=1 SV=6
7	0	6	27.3800	17.2600	17.2600	tr|E9PI65|E9PI65_HUMAN	Heat shock cognate 71 kDa protein (Fragment) OS=*Homo sapiens* GN=HSPA8 PE=1 SV=1
7	0	4.05	9.5690	3.9870	3.9870	tr|E9PKE3|E9PKE3_HUMAN	Heat shock cognate 71 kDa protein OS=*Homo sapiens* GN=HSPA8 PE=1 SV=1
8	3.02	3.02	6.8970	6.8970	6.8970	tr|E7EQ64|E7EQ64_HUMAN	Trypsin-1 OS=*Homo sapiens* GN=PRSS1 PE=1 SV=1
8	0	3.02	7.5950	7.5950	7.5950	tr|A6XGL3|A6XGL3_HUMAN	Protease serine 1 OS=*Homo sapiens* GN=PRSS1 PE=1 SV=1
8	0	3.02	7.2870	7.2870	7.2870	sp|P07477|TRY1_HUMAN	Trypsin-1 OS=*Homo sapiens* GN=PRSS1 PE=1 SV=1
8	0	1.34	7.0420	7.0420	7.0420	tr|H0Y8D1|H0Y8D1_HUMAN	Trypsin-1 (Fragment) OS=*Homo sapiens* GN=PRSS1 PE=1 SV=1
8	0	1.34	4.0490	4.0490	4.0490	tr|A0A087WW55|A0A087WW55_HUMAN	Trypsin-1 OS=*Homo sapiens* GN=PRSS1 PE=1 SV=1
8	0	1.33	1.3650	1.3650	1.3650	tr|J3KQC6|J3KQC6_HUMAN	Transmembrane protease serine 13 OS=*Homo sapiens* GN=TMPRSS13 PE=3 SV=1
8	0	1.33	1.4110	1.4110	1.4110	tr|E9PRA0|E9PRA0_HUMAN	Transmembrane protease serine 13 OS=*Homo sapiens* GN=TMPRSS13 PE=3 SV=1
8	0	1.33	1.4230	1.4230	1.4230	tr|A0A087WWD8|A0A087WWD8_HUMAN	Transmembrane protease serine 13 OS=*Homo sapiens* GN=TMPRSS13 PE=3 SV=1
8	0	1.33	1.3650	1.3650	1.3650	sp|Q9BYE2|TMPSD_HUMAN	Transmembrane protease serine 13 OS=*Homo sapiens* GN=TMPRSS13 PE=2 SV=4
9	2.6	2.6	11.1700	4.2550	0.9309	tr|A0A0C4DGX4|A0A0C4DGX4_HUMAN	Cullin-1 OS=*Homo sapiens* GN=CUL1 PE=1 SV=1
9	0	2.6	10.8200	4.1240	0.9021	sp|Q13616|CUL1_HUMAN	Cullin-1 OS=*Homo sapiens* GN=CUL1 PE=1 SV=2
10	2	6.11	6.8640	6.2400	6.2400	tr|Q53FA3|Q53FA3_HUMAN	Heat shock 70 kDa protein 1-like (Fragment) OS=*Homo sapiens* GN=HSPA1L PE=1 SV=1
10	0	6.11	6.8540	6.2310	6.2310	tr|A0A0G2JIW1|A0A0G2JIW1_HUMAN	Heat shock 70 kDa protein 1B OS=*Homo sapiens* GN=HSPA1B PE=1 SV=1
10	0	6.11	6.8640	6.2400	6.2400	sp|P34931|HS71L_HUMAN	Heat shock 70 kDa protein 1-like OS=*Homo sapiens* GN=HSPA1L PE=1 SV=2
10	0	6.11	6.8640	6.2400	6.2400	sp|P0DMV9|HS71B_HUMAN	Heat shock 70 kDa protein 1B OS=*Homo sapiens* GN=HSPA1B PE=1 SV=1
10	0	6.11	6.8640	6.2400	6.2400	sp|P0DMV8|HS71A_HUMAN	Heat shock 70 kDa protein 1A OS=*Homo sapiens* GN=HSPA1A PE=1 SV=1
11	2	2	11.5000	3.5210	3.5210	tr|Q5JP53|Q5JP53_HUMAN	Tubulin beta chain OS=*Homo sapiens* GN=TUBB PE=1 SV=1
11	0	2	11.0400	3.3780	3.3780	sp|P07437|TBB5_HUMAN	Tubulin beta chain OS=*Homo sapiens* GN=TUBB PE=1 SV=2
11	0	2	9.1400	4.0320	4.0320	tr|Q5ST81|Q5ST81_HUMAN	Tubulin beta chain OS=*Homo sapiens* GN=TUBB PE=1 SV=1
12	2	2	5.6690	2.4940	2.4940	tr|A0A087WVQ9|A0A087WVQ9_HUMAN	Elongation factor 1-alpha 1 OS=*Homo sapiens* GN=EEF1A1 PE=1 SV=1
12	0	2	5.4110	2.3810	2.3810	sp|Q5VTE0|EF1A3_HUMAN	Putative elongation factor 1-alpha-like 3 OS=*Homo sapiens* GN=EEF1A1P5 PE=5 SV=1
12	0	2	5.4110	2.3810	2.3810	sp|P68104|EF1A1_HUMAN	Elongation factor 1-alpha 1 OS=*Homo sapiens* GN=EEF1A1 PE=1 SV=1
12	0	2	3.8880	2.3760	2.3760	sp|Q05639|EF1A2_HUMAN	Elongation factor 1-alpha 2 OS=*Homo sapiens* GN=EEF1A2 PE=1 SV=1
13	2	2	19.6700	17.0500	17.0500	tr|Q96C32|Q96C32_HUMAN	Polyubiquitin-C OS=*Homo sapiens* GN=UBC PE=1 SV=1
13	0	2	19.6500	17.0300	17.0300	tr|Q5PY61|Q5PY61_HUMAN	Polyubiquitin-C OS=*Homo sapiens* GN=UBC PE=1 SV=1
13	0	2	10.8700	9.4200	9.4200	tr|M0R2S1|M0R2S1_HUMAN	Ubiquitin-60S ribosomal protein L40 (Fragment) OS=*Homo sapiens* GN=UBA52 PE=1 SV=1
13	0	2	23.8100	20.6300	20.6300	tr|M0R1V7|M0R1V7_HUMAN	Ubiquitin-60S ribosomal protein L40 (Fragment) OS=*Homo sapiens* GN=UBA52 PE=1 SV=1
13	0	2	13.1600	11.4000	11.4000	tr|M0R1M6|M0R1M6_HUMAN	Ubiquitin-60S ribosomal protein L40 (Fragment) OS=*Homo sapiens* GN=UBA52 PE=1 SV=1
13	0	2	28.3000	24.5300	24.5300	tr|K7EMA8|K7EMA8_HUMAN	Protein UBBP4 (Fragment) OS=*Homo sapiens* GN=UBBP4 PE=1 SV=1
13	0	2	14.1500	12.2600	12.2600	tr|J3QTR3|J3QTR3_HUMAN	Ubiquitin-40S ribosomal protein S27a (Fragment) OS=*Homo sapiens* GN=RPS27 A PE=1 SV=1
13	0	2	34.8800	30.2300	30.2300	tr|J3QSA3|J3QSA3_HUMAN	Polyubiquitin-B (Fragment) OS=*Homo sapiens* GN=UBB PE=1 SV=1
13	0	2	16.1300	13.9800	13.9800	tr|J3QS39|J3QS39_HUMAN	Polyubiquitin-B (Fragment) OS=*Homo sapiens* GN=UBB PE=1 SV=1
13	0	2	6.5500	5.6770	5.6770	tr|J3QRK5|J3QRK5_HUMAN	Protein UBBP4 OS=*Homo sapiens* GN=UBBP4 PE=1 SV=1
13	0	2	6.6960	5.8040	5.8040	tr|J3QLP7|J3QLP7_HUMAN	Protein UBBP4 OS=*Homo sapiens* GN=UBBP4 PE=1 SV=1
13	0	2	21.8400	18.9300	18.9300	tr|J3QKN0|J3QKN0_HUMAN	Polyubiquitin-B (Fragment) OS=*Homo sapiens* GN=UBB PE=1 SV=1
13	0	2	18.7500	16.2500	16.2500	tr|F5H747|F5H747_HUMAN	Polyubiquitin-C (Fragment) OS=*Homo sapiens* GN=UBC PE=1 SV=6
13	0	2	24.5900	21.3100	21.3100	tr|F5H6Q2|F5H6Q2_HUMAN	Polyubiquitin-C (Fragment) OS=*Homo sapiens* GN=UBC PE=1 SV=6
13	0	2	19.3500	16.7700	16.7700	tr|F5H388|F5H388_HUMAN	Polyubiquitin-C (Fragment) OS=*Homo sapiens* GN=UBC PE=1 SV=1
13	0	2	22.0600	19.1200	19.1200	tr|F5H2Z3|F5H2Z3_HUMAN	Polyubiquitin-C (Fragment) OS=*Homo sapiens* GN=UBC PE=1 SV=1
13	0	2	20.1300	17.4500	17.4500	tr|F5H265|F5H265_HUMAN	Polyubiquitin-C (Fragment) OS=*Homo sapiens* GN=UBC PE=1 SV=1
13	0	2	24.5900	21.3100	21.3100	tr|F5GZ39|F5GZ39_HUMAN	Polyubiquitin-C (Fragment) OS=*Homo sapiens* GN=UBC PE=4 SV=1
13	0	2	22.3900	19.4000	19.4000	tr|F5GYU3|F5GYU3_HUMAN	Polyubiquitin-C (Fragment) OS=*Homo sapiens* GN=UBC PE=1 SV=1
13	0	2	17.7500	15.3800	15.3800	tr|F5GXK7|F5GXK7_HUMAN	Polyubiquitin-C (Fragment) OS=*Homo sapiens* GN=UBC PE=1 SV=2
13	0	2	19.6100	16.9900	16.9900	tr|B4DV12|B4DV12_HUMAN	Polyubiquitin-B OS=*Homo sapiens* GN=UBB PE=1 SV=1
13	0	2	11.7200	10.1600	10.1600	sp|P62987|RL40_HUMAN	Ubiquitin-60S ribosomal protein L40 OS=*Homo sapiens* GN=UBA52 PE=1 SV=2
13	0	2	9.6150	8.3330	8.3330	sp|P62979|RS27A_HUMAN	Ubiquitin-40S ribosomal protein S27a OS=*Homo sapiens* GN=RPS27 A PE=1 SV=2
13	0	2	19.7100	17.0800	17.0800	sp|P0CG48|UBC_HUMAN	Polyubiquitin-C OS=*Homo sapiens* GN=UBC PE=1 SV=3
13	0	2	19.6500	17.0300	17.0300	sp|P0CG47|UBB_HUMAN	Polyubiquitin-B OS=*Homo sapiens* GN=UBB PE=1 SV=1
14	2	2	6.1370	6.1370	6.1370	tr|H0YB39|H0YB39_HUMAN	Heterogeneous nuclear ribonucleoprotein H (Fragment) OS=*Homo sapiens* GN=HNRNPH1 PE=1 SV=1
14	0	2	3.6020	3.6020	3.6020	tr|G8JLB6|G8JLB6_HUMAN	Heterogeneous nuclear ribonucleoprotein H OS=*Homo sapiens* GN=HNRNPH1 PE=1 SV=1
14	0	2	3.9630	3.9630	3.9630	tr|E9PCY7|E9PCY7_HUMAN	Heterogeneous nuclear ribonucleoprotein H OS=*Homo sapiens* GN=HNRNPH1 PE=1 SV=1
14	0	2	10.2400	10.2400	10.2400	tr|E7EN40|E7EN40_HUMAN	Heterogeneous nuclear ribonucleoprotein H (Fragment) OS=*Homo sapiens* GN=HNRNPH1 PE=1 SV=1
14	0	2	10.9700	10.9700	10.9700	tr|E5RGV0|E5RGV0_HUMAN	Heterogeneous nuclear ribonucleoprotein H (Fragment) OS=*Homo sapiens* GN=HNRNPH1 PE=1 SV=1
14	0	2	17.0000	17.0000	17.0000	tr|E5RGH4|E5RGH4_HUMAN	Heterogeneous nuclear ribonucleoprotein H (Fragment) OS=*Homo sapiens* GN=HNRNPH1 PE=1 SV=1
14	0	2	10.1200	10.1200	10.1200	tr|D6RIU0|D6RIU0_HUMAN	Heterogeneous nuclear ribonucleoprotein H (Fragment) OS=*Homo sapiens* GN=HNRNPH1 PE=1 SV=1
14	0	2	8.0190	8.0190	8.0190	tr|D6RBM0|D6RBM0_HUMAN	Heterogeneous nuclear ribonucleoprotein H (Fragment) OS=*Homo sapiens* GN=HNRNPH1 PE=1 SV=1
14	0	2	3.7860	3.7860	3.7860	sp|P55795|HNRH2_HUMAN	Heterogeneous nuclear ribonucleoprotein H2 OS=*Homo sapiens* GN=HNRNPH2 PE=1 SV=1
14	0	2	3.7860	3.7860	3.7860	sp|P31943|HNRH1_HUMAN	Heterogeneous nuclear ribonucleoprotein H OS=*Homo sapiens* GN=HNRNPH1 PE=1 SV=4
15	2	2	20.0000	20.0000	20.0000	tr|F8VX09|F8VX09_HUMAN	Tubulin alpha-1B chain (Fragment) OS=*Homo sapiens* GN=TUBA1B PE=1 SV=1
15	0	2	18.5200	18.5200	18.5200	tr|F8VWV9|F8VWV9_HUMAN	Tubulin alpha-1B chain (Fragment) OS=*Homo sapiens* GN=TUBA1B PE=1 SV=1
15	0	2	6.0980	6.0980	6.0980	tr|F8VVB9|F8VVB9_HUMAN	Tubulin alpha-1B chain (Fragment) OS=*Homo sapiens* GN=TUBA1B PE=1 SV=6
15	0	2	11.5400	11.5400	11.5400	tr|F8VS66|F8VS66_HUMAN	Tubulin alpha-1C chain OS=*Homo sapiens* GN=TUBA1C PE=4 SV=1
15	0	2	13.3900	13.3900	13.3900	tr|F8VRZ4|F8VRZ4_HUMAN	Tubulin alpha-1A chain (Fragment) OS=*Homo sapiens* GN=TUBA1A PE=4 SV=1
15	0	2	30.6100	30.6100	30.6100	tr|F8VRK0|F8VRK0_HUMAN	Tubulin alpha-1B chain (Fragment) OS=*Homo sapiens* GN=TUBA1B PE=1 SV=1
15	0	2	6.8490	6.8490	6.8490	tr|F8VQQ4|F8VQQ4_HUMAN	Tubulin alpha-1A chain (Fragment) OS=*Homo sapiens* GN=TUBA1A PE=1 SV=1
15	0	2	2.8900	2.8900	2.8900	tr|F5H5D3|F5H5D3_HUMAN	Tubulin alpha-1C chain OS=*Homo sapiens* GN=TUBA1C PE=1 SV=1
15	0	2	3.3410	3.3410	3.3410	sp|Q9BQE3|TBA1C_HUMAN	Tubulin alpha-1C chain OS=*Homo sapiens* GN=TUBA1C PE=1 SV=1
15	0	2	3.3260	3.3260	3.3260	sp|Q71U36|TBA1A_HUMAN	Tubulin alpha-1A chain OS=*Homo sapiens* GN=TUBA1A PE=1 SV=1
15	0	2	3.3260	3.3260	3.3260	sp|P68363|TBA1B_HUMAN	Tubulin alpha-1B chain OS=*Homo sapiens* GN=TUBA1B PE=1 SV=1
16	2	2	3.7040	3.7040	3.7040	tr|F8VVM2|F8VVM2_HUMAN	Phosphate carrier protein, mitochondrial OS=*Homo sapiens* GN=SLC25A3 PE=1 SV=1
16	0	2	3.3150	3.3150	3.3150	sp|Q00325|MPCP_HUMAN	Phosphate carrier protein, mitochondrial OS=*Homo sapiens* GN=SLC25A3 PE=1 SV=2
17	0.91	0.99	4.9150	3.8980	1.8640	sp|P13647|K2C5_HUMAN	Keratin, type II cytoskeletal 5 OS=*Homo sapiens* GN=KRT5 PE=1 SV=3
17	0	0.91	8.3330	8.3330	8.3330	tr|F8VV57|F8VV57_HUMAN	Keratin, type II cytoskeletal 5 (Fragment) OS=*Homo sapiens* GN=KRT5 PE=1 SV=1
18	0.75	0.75	5.7690	5.7690	5.7690	tr|E7EUT5|E7EUT5_HUMAN	Glyceraldehyde-3-phosphate dehydrogenase OS=*Homo sapiens* GN=GAPDH PE=1 SV=1
18	0	0.75	4.4780	4.4780	4.4780	sp|P04406|G3P_HUMAN	Glyceraldehyde-3-phosphate dehydrogenase OS=*Homo sapiens* GN=GAPDH PE=1 SV=3
19	0.41	0.41	3.7210	3.7210	0.0000	RRRRRtr|I3L3P0|I3L3P0_HUMAN	REVERSED Membrane-associated tyrosine- and threonine-specific cdc2-inhibitory kinase (Fragment) OS=*Homo sapiens* GN=PKMYT1 PE=1 SV=1
20	0.11	0.11	5.4790	5.4790	0.0000	RRRRRtr|J3QS16|J3QS16_HUMAN	REVERSED Uncharacterized protein C19orf47 (Fragment) OS=*Homo sapiens* GN=C19orf47 PE=1 SV=1
20	0	0.11	5.6340	5.6340	0.0000	RRRRRtr|J3QKZ5|J3QKZ5_HUMAN	REVERSED Uncharacterized protein C19orf47 (Fragment) OS=*Homo sapiens* GN=C19orf47 PE=1 SV=1
20	0	0.11	1.8960	1.8960	0.0000	RRRRRsp|Q8N9M1|CS047_HUMAN	REVERSED Uncharacterized protein C19orf47 OS=*Homo sapiens* GN=C19orf47 PE=1 SV=1
21	0.05	0.05	1.7280	1.7280	0.0000	RRRRRtr|A0A0C4DGG1|A0A0C4DGG1_HUMAN	REVERSED Protein kinase C and casein kinase substrate in neurons protein 3 (Fragment) OS=*Homo sapiens* GN=PACSIN3 PE=1 SV=1
21	0	0.05	1.6510	1.6510	0.0000	RRRRRsp|Q9UKS6|PACN3_HUMAN	REVERSED Protein kinase C and casein kinase substrate in neurons protein 3 OS=*Homo sapiens* GN=PACSIN3 PE=1 SV=2

### Overexpression of SLC25A3 could negatively regulate NLRP3 inflammasome activation

**Figure 2:**
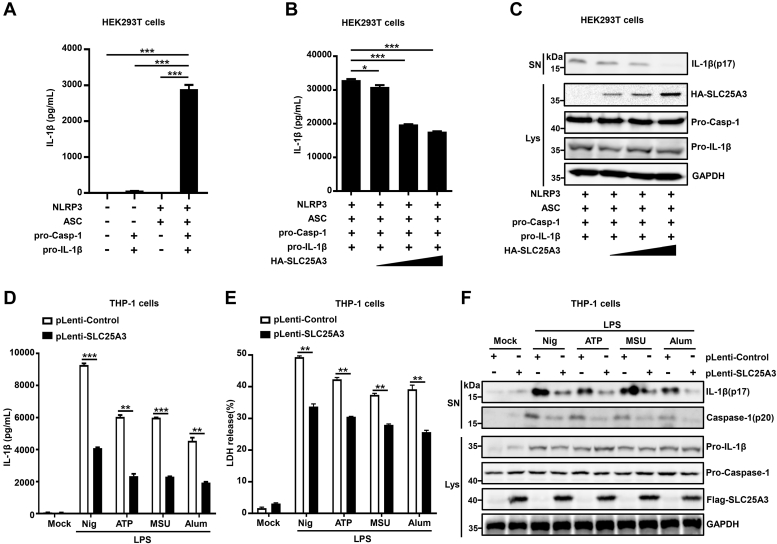
**Overexpression of SLC25A3 could negatively regulate NLRP3 inflammasome activation.***A*, HEK293T cells were transfected with Vector, Vector-caspase-1 and Vector-IL-1β, Vector-NLRP3 and Vector-ASC, or Vector-NLRP3, Vector-ASC, Vector-caspase-1, and Vector-IL-1β as indicated in the figure. Secreted IL-1β in the supernatants was analyzed by ELISA. *B* and *C*, HEK293T cells were cotransfected with Vector-NLRP3, Vector-ASC, Vector-caspase-1, and Vector-ASC four plasmids, along with a series of Vector-HA-SLC25A3. Secreted IL-1β in the supernatants were analyzed by ELISA (*B*). Matured IL-1β (p17) in the cell supernatants and the cell lysates were immunoprecipitated with indicated antibodies (*C*). *D*–*F*, THP-1 derived macrophages stably expressing pLenti-control or pLenti-SLC25A3 were primed with LPS (100 ng/ml) for 3 h, stimulated by ATP (5 mM) for 1 h, nigericin (10 μM) for 1 h, MSU (500 μg/ml) for 2 h, Alum (400 μg/ml) for 6 h. Secreted IL-1β in the supernatants were analyzed by ELISA (*D*). The release of LDH in the supernatants were measured by cytotoxicity assay (*E*). Matured IL-1β (p17) and matured caspase-1 (p20) in the supernatants and the cell lysates were determined by immunoblot analysis with indicated antibodies (*F*). Data shown are mean ± SEM, ∗*p* < 0.05, ∗∗*p* < 0.01, ∗∗∗*p* < 0.0001. IL-1β, interleukin 1β; LDH, lactate dehydrogenase; LPS, lipopolysaccharide; MSU, monosodium urate; NLRP3, NACHT, leucine-rich repeat, and pyrin domain-containing protein 3; SLC25A3, solute carrier family 25 member 3.

To reveal whether SLC25A3 could regulate NLRP3 inflammasome activation, we constructed a reconstructed NLRP3 inflammasome model, in which HEK293T cells were cotransfected with four plasmids encoding NLRP3, ASC, pro-caspase-1, and pro-IL-1β proteins. Reconstructed NLRP3 inflammasome model was constructed successfully by detecting the secretion of mature IL-1β (Fig 2*A*). IL-1β secretion (Fig. 2*B*), and mature IL-1β or mature Casp-1 in the supernatant (Fig. 2*C*) was inhibited by SLC25A3 in reconstructed NLRP3 inflammasome model, and the inhibitory effect is dependent with the plasmid concentration of SLC25A3. To further reveal the role of SLC25A3 in repression of endogenous NLRP3 inflammasome activation, we constructed THP-1 cell lines stably overexpressing SLC25A3. The THP-1 cell lines stably expressing control or SLC25A3 were differentiated into macrophages and stimulated with lipopolysaccharide (LPS) plus nigericin, LPS plus ATP, LPS plus MSU, or LPS plus Alum. IL-1β secretion (Fig. 2*D*), the release of lactate dehydrogenase (LDH) in the supernatant (Fig. 2*E*), and mature IL-1β and mature Casp-1 in the supernatant (Fig. 2*F*) were significantly attenuated by SLC25A3. Thus, we uncovered that SLC25A3 suppressed NLRP3 inflammasome activation.

### Knockdown of SLC25A3 could promote NLRP3 inflammasome activation

**Figure 3:**
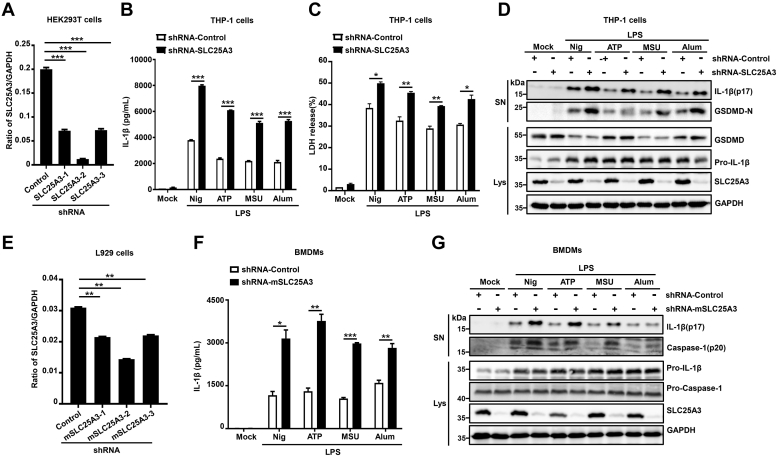
**Knockdown of SLC25A3 could promote NLRP3 inflammasome activation.***A*, HEK293T cells were infected with plasmids stably expressing shRNA-control, shRNA-SLC25A3-1, shRNA-SLC25A3-2, or shRNA-SLC25A3-3. SLC25A3 and GAPDH mRNAs were determined by quantitative RT-PCR. *B*–*D*, THP-1-derived macrophages stably expressing shRNA-control or shRNA-SLC25A3 were treated with and primed with LPS (100 ng/ml) for 3 h, stimulated by ATP (5 mM) for 1 h, nigericin (10 μM) for 1 h, MSU (500 μg/ml) for 2 h, and Alum (400 μg/ml) for 6 h. Secreted IL-1β in the supernatants were analyzed by ELISA (*B*). The release of LDH in the supernatants were measured by cytotoxicity assay (*C*). Matured IL-1β (p17) in the supernatants and the cell lysates were determined by immunoblot analysis with indicated antibodies (*D*). *E*, L929 cells were transfected with Vector-shRNA-control, Vector-shRNA-mSLC25A3-1, Vector-shRNA-mSLC25A3-2, Vector-shRNA-mSLC25A3-3, SLC25A3, and GAPDH mRNAs were determined by quantitative RT-PCR. *F*-*G*, BMDMs stably expressing shRNA-control or shRNA-mSLC25A3 were primed with LPS (100 ng/ml) for 3 h, stimulated by ATP (5 mM) for 1 h, nigericin (10 μM) for 1 h, MSU (500 μg/ml) for 2 h, Alum (400 μg/ml) for 6 h. Secreted IL-1β in the supernatants were analyzed by ELISA (*F*). Matured IL-1β (p17) and matured caspase-1 (p20) in the supernatants and the cell lysates were determined by immunoblot analysis with indicated antibodies (*G*). Data shown are mean ± SEM, ∗*p* < 0.05, ∗∗*p* < 0.01, ∗∗∗*p* < 0.0001. BMDM, bone marrow-derived macrophage; IL-1β, interleukin 1β; LDH, lactate dehydrogenase; LPS, lipopolysaccharide; MSU, monosodium urate; NLRP3, NACHT, leucine-rich repeat, and pyrin domain-containing protein 3; SLC25A3, solute carrier family 25 member 3.

To further reveal whether SLC25A3 could regulate NLRP3 inflammasome activation, we constructed plasmids stably overexpressing shRNA-control, shRNA-SLC25A3-1, shRNA-SLC25A3-2, or shRNA-SLC25A3-3. HEK293T cells were transfected with these vectors. The result indicated that knockdown effect of shRNA-SLC25A3-2 is the best by RT-PCR (Fig. 3*A*). THP-1 cell lines stably expressing shRNA-control or shRNA-SLC25A3 were differentiated into macrophages and stimulated with LPS plus nigericin, LPS plus ATP, LPS plus MSU, or LPS plus Alum. IL-1β secretion (Fig. 3*B*), the release of LDH in the supernatant (Fig. 3*C*), and mature IL-1β and the cleavage of GSDMD in the supernatant (Fig. 3*D*) were obviously reduced by SLC25A3. To further uncover the function of SLC25A3 in NLRP3 inflammasome activation in primary cells, we also constructed four vectors (shRNA-control, shRNA-mSLC25A3-1, shRNA-mSLC25A3-2, and mSLC25A3-3). In L929 cells, we validated the effect of these vectors. The result indicated that knockdown effect of shRNA-mSLC25A3-2 is the best (Fig. 3*E*). BMDMs were infected with lentivirus containing shRNA-control vector or shRNA-mSLC25A3, subsequently stimulated by LPS plus nigericin, LPS plus ATP, LPS plus MSU, and LPS plus Alum. The results indicated that IL-1β secretion (Fig. 3*F*), and mature IL-1β and mature Casp-1 in the supernatant (Fig. 3*G*) were also markedly reduced by mSLC25A3. Overall, we found that knockdown of SLC25A3 could promote NLRP3 inflammasome activation.

### SLC25A3 specifically suppressed NLRP3 inflammasome activation

**Figure 4:**
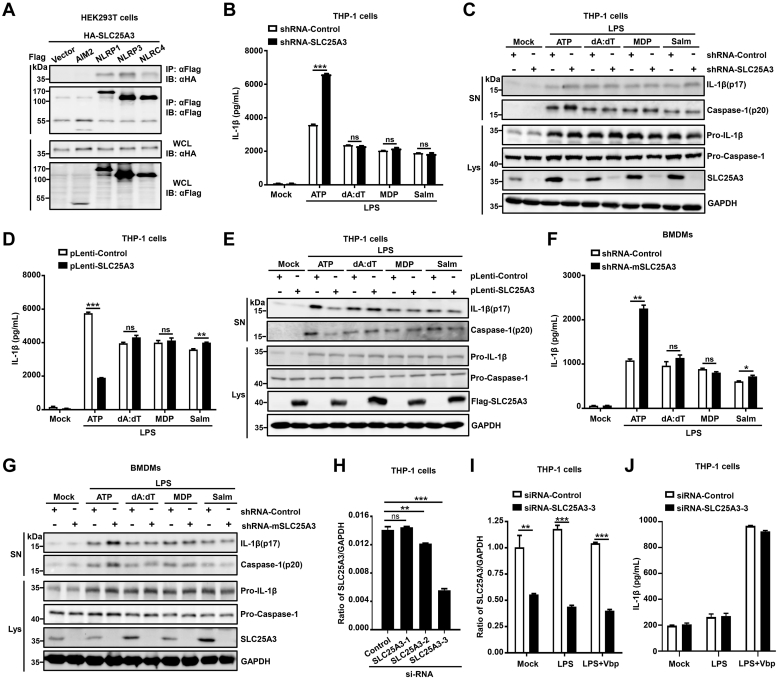
**SLC25A3 specifically suppressed NLRP3 inflammasome activation.***A*, HEK293T cells were transfected with HA-SLC25A3 and Vector-Flag, Flag-AIM2, Flag-NLRP1, Flag-NLRP3, or Flag-NLRC4 as indicated in the figure. The cell lysates were immunoprecipitated with anti-Flag antibody and then immunoblotted with indicated antibodies. *B* and *C*, THP-1 derived macrophages stably expressing shRNA-control or shRNA-SLC25A3 were treated and primed with LPS (100 ng/ml) for 3 h, stimulated by ATP (5 mM) for 1 h, Poly(dA:dT) (1 μg/ml) for 12 h, MDP (100 μg/ml) for 12 h, *Salmonella* typhimurium (MOI = 2) for 12 h. Secreted IL-1β in the supernatants were analyzed by ELISA (*B*). Matured IL-1β (p17) and matured Caspase-1 (p20) in the supernatants and the cell lysates were determined by immunoblot analysis with indicated antibodies (*C*). *D* and *E*, THP-1 derived macrophages stably expressing pLenti-control or pLenti-SLC25A3 were primed with LPS (100 ng/ml) for 3 h, stimulated by ATP (5 mM) for 1 h, Poly(dA:dT) (1 μg/ml) for 12 h, MDP (100 μg/ml) for 12 h, *Salmonella typhimurium* (MOI = 2) for 12 h. Secreted IL-1β in the supernatants was analyzed by ELISA (*D*). Matured IL-1β (p17) and matured caspase-1 (p20) in the supernatants and the cell lysates were determined by immunoblot analysis with indicated antibodies (*E*). *F* and *G*, BMDMs infected by lentiviruses stably expressing shRNA-control or shRNA-mSLC25A3 were primed with LPS (100 ng/ml) for 3 h, stimulated by ATP (5 mM) for 1 h, Poly(dA:dT) (1 μg/ml) for 12 h, MDP (100 μg/ml) for 12 h, *Salmonella typhimurium* (MOI = 2) for 12 h. Secreted IL-1β in the supernatants was analyzed by ELISA (*F*). Matured IL-1β (p17) and matured caspase-1 (p20) in the supernatants and the cell lysates were determined by immunoblot analysis with indicated antibodies (*G*). *H*–*J*, THP-1-derived macrophages were transfected with four siRNAs (siRNA-NC, siRNA-SLC25A3-1, siRNA-SLC25A3-2, and siRNA-SLC25A3-3). SLC25A3 and GAPDH RNAs were determined by quantitative RT-PCR (*H*). THP-1-derived macrophages were stimulated by LPS (100 ng/ml, 3 h) or LPS (100 ng/ml, 3 h) plus Val-boroPro(20 μM, 8 h). SLC25A3 and GAPDH RNAs were determined by quantitative RT-PCR (*I*). Secreted IL-1β in the supernatants was analyzed by ELISA (*J*). Data shown are mean ± SEM, ∗*p* < 0.05, ∗∗*p* < 0.01, ∗∗∗*p* < 0.0001. BMDM, bone marrow-derived macrophage; LPS, lipopolysaccharide; MDP, muramyl dipeptide; MOI, multiplicity of infection; NLRP3, NACHT, leucine-rich repeat, and pyrin domain-containing protein 3; SLC25A3, solute carrier family 25 member 3.

**Supporting Figure S1:**
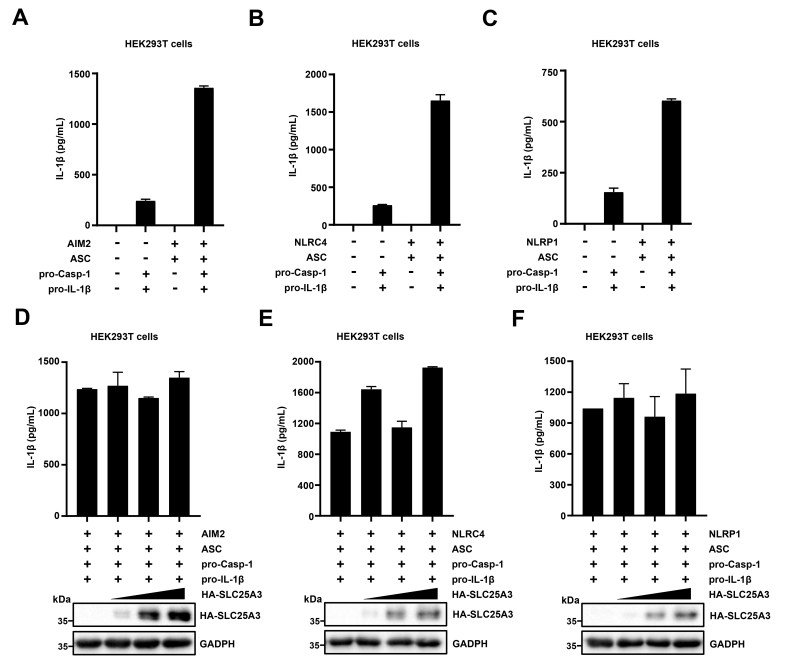


To reveal whether SLC25A3 specifically suppressed NLRP3 inflammasome activation, we would explore its function in other inflammasomes (NLRP1, NLRC4, and AIM2 inflammasomes). SLC25A3 could interact with NLRP3, NLRP3, or NLRC4, but not with AIM2 (Fig 4*A*). We also constructed a reconstructed AIM2/NLRC4/NLRP1 inflammasome model, in which HEK293T cells were cotransfected with four plasmids encoding AIM2/NLRC4/NLRP1, ASC, pro-caspase-1, and pro-IL-1β proteins. The reconstructed AIM2/NLRC4/NLRP1 inflammasome model was successfully constructed by detecting the secretion of mature IL-1β (Fig. S1, *A*–*C*). IL-1β secretion was not inhibited by SLC25A3 in reconstructed AIM2/NLRC4/NLRP1 inflammasome model, and the content of IL-1β secretion was independent with the plasmid concentration of SLC25A3 (Fig. S1, *D*–*F*). THP-1 cell lines stably expressing shRNA-control or shRNA-SLC25A3 were differentiated into macrophages and stimulated with LPS plus ATP, LPS plus poly(dA:dT), LPS plus muramyl dipeptide (MDP), or LPS plus Salm. IL-1β secretion (Fig. 4*B*), and mature IL-1β and mature Casp-1 in the supernatant (Fig. 4*C*) were obviously reduced by ATP (a stimulator of NLRP3 inflammasome), but not by poly(dA:dT) (a stimulator of AIM2 inflammasome), MDP (a stimulator of NLRP1 inflammasome), or Salm (a stimulator of NLRC4 inflammasome). These results indicated that knockdown of SLC25A3 could specifically promote NLRP3 inflammasome activation. THP-1 cell lines stably expressing plenti-control or plenti-SLC25A3 were differentiated into macrophages and stimulated with LPS plus ATP, LPS plus poly(dA:dT), LPS plus MDP, or LPS plus Salm. IL-1β secretion (Fig. 4*D*) and mature IL-1β and mature Casp-1 in the supernatant (Fig. 4*E*) were significantly enhanced by LPS plus ATP, but not by LPS plus poly(dA:dT), LPS plus MDP, or LPS plus Salm. BMDMs were infected with lentivirus containing shRNA-control vector or shRNA-mSLC25A3, subsequently stimulated by LPS plus ATP, LPS plus poly(dA:dT), LPS plus MDP, or LPS plus Salm. The results indicated that IL-1β secretion (Fig. 4*F*), and mature IL-1β and mature Casp-1 in the supernatant (Fig. 4*G*) were also markedly enhanced by LPS plus ATP, but not by LPS plus poly(dA:dT), LPS plus MDP, or LPS plus Salm. Knockdown of SLC25A3 could specifically enhance NLRP3 inflammasome activation in BMDMs. However, for the THP-1 cells, poly(dA:dT) and Salm is good to activate AIM2 and NLRC4 inflammasome, MDP was not suitable to activate NLRP1 inflammasome, and it was more widely known as a activator of NOD2. To verify the accuracy of the above results, Vbp (Val-boroPro) was used as a stimulus for NLRP1 inflammasome. Three siRNA-SLC25A3(-1, -2, -3) were identified by RT-PCR in THP-1 cells, and the effect of siRNA-SLC25A3-3 specific to SLC25A3 was the best (Fig. 4*H*). THP-1 derived macrophages were transfected with siRNA-control or siRNA-SLC25A3-3 and then stimulated with LPS, or LPS plus Val. The result indicated that knockdown effect of siRNA-SLC25A3-3 in the cells were effective (Fig. 4*I*). Vbp could indeed activate NLRP1 inflammasome by measuring the content of IL-1β secretion (Fig. 4*J*). However, knockdown of SLC25A3 could not enhance NLRP1 inflammasome activation stimulated by LPS plus Vbp (Fig. 4*J*). Overall, we found that SLC25A3 could specifically suppress NLRP3 inflammasome activation.

### SLC25A3 suppressed ASC oligomerization mediated by NLRP3 inflammasome

**Figure 5:**
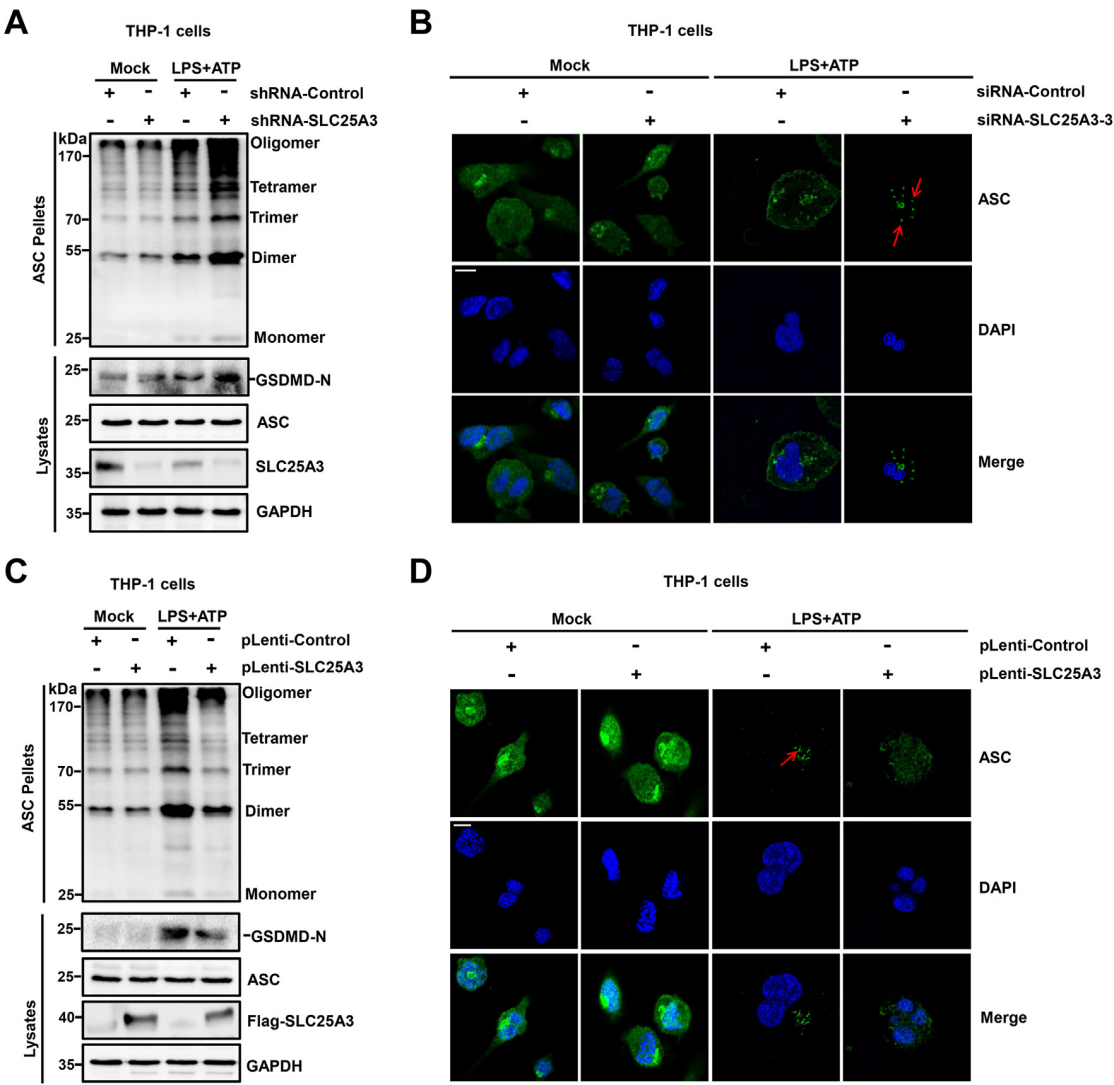
**SLC25A3 suppressed ASC oligomerization mediated by NLRP3 inflammasome.***A*, THP-1-derived macrophages stably expressing shRNA-control or shRNA-SLC25A3 were treated with LPS (100 ng/ml) for 3 h, stimulated by ATP (5 mM) for 1 h. The cell lysates were determined by immunoblot analysis with indicated antibodies. *B*, THP-1 derived macrophages transfected with siRNA-control or siRNA-SLC25A3-3 were treated with LPS (100 ng/ml) for 3 h, stimulated by ATP (5 mM) for 30 min. The cells were immunostained with anti-ASC. Subcellular localizations of ACS (*green*) and the nucleus marker DAPI (*blue*) were examined under confocal microscopy. The scale bar represents 10 μm. *C*, THP-1 derived macrophages stably expressing pLenti-control or pLenti-SLC25A3 were treated with LPS (100 ng/ml) for 3 h, stimulated by ATP (5 mM) for 1 h. The cell lysates were determined by immunoblot analysis with indicated antibodies. *D*, THP-1 derived macrophages stably expressing pLenti-control or pLenti-SLC25A3 were treated with LPS (100 ng/ml) for 3 h, stimulated by ATP (5 mM) for 30 min. The cells were immunostained with anti-ASC. Subcellular localizations of and ACS (*green*) and the nucleus marker DAPI (*blue*) were examined under confocal microscopy. The scale bar represents 10 μm. DAPI, 4′,6-diamidino-2-phenylindole; LPS, lipopolysaccharide; NLRP3, NACHT, leucine-rich repeat, and pyrin domain-containing protein 3; SLC25A3, solute carrier family 25 member 3.

SLC25A3 could inhibit mature IL-1β and Casp-1 mediated by NLRP3 inflammasome activation. To further uncover how SLC25A3 negatively regulates NLRP3 inflammasome activation. THP-1 cell lines stably expressing shRNA-control or shRNA-SLC25A3 were differentiated into macrophages, and subsequently stimulated with LPS plus ATP. The result indicated that knockdown of SLC25A3 promoted ASC oligomerization and the cleavage of GSDMD in the cells (Fig. 5*A*). Furthermore, the speck formation of endogenous ASC was enhanced by LPS plus ATP in the cells transfected with siRNA-SLC25A3-3 compared to the cells transfected with siRNA-control (Fig. 5*B*). THP-1 cell lines stably expressing plenti-control or plenti-SLC25A3 were differentiated into macrophages, and subsequently stimulated with LPS plus ATP. The result demonstrated that overexpressing of SLC25A3 promoted ASC oligomerization and the cleavage of GSDMD in the cells (Fig. 5*C*). Also, the speck formation of endogenous ASC was attenuated by LPS plus ATP in the cells stably expressing plenti-SLC25A3 compared to the cells stably expressing plenti-control (Fig. 5*D*). Taken together, we found that SLC25A3 suppressed ASC oligomerization mediated by NLRP3 inflammasome.

### The interaction of SLC25A3 and NLRP3 was enhanced by NLRP3 inflammasome inducers in the mitochondria

**Figure 6:**
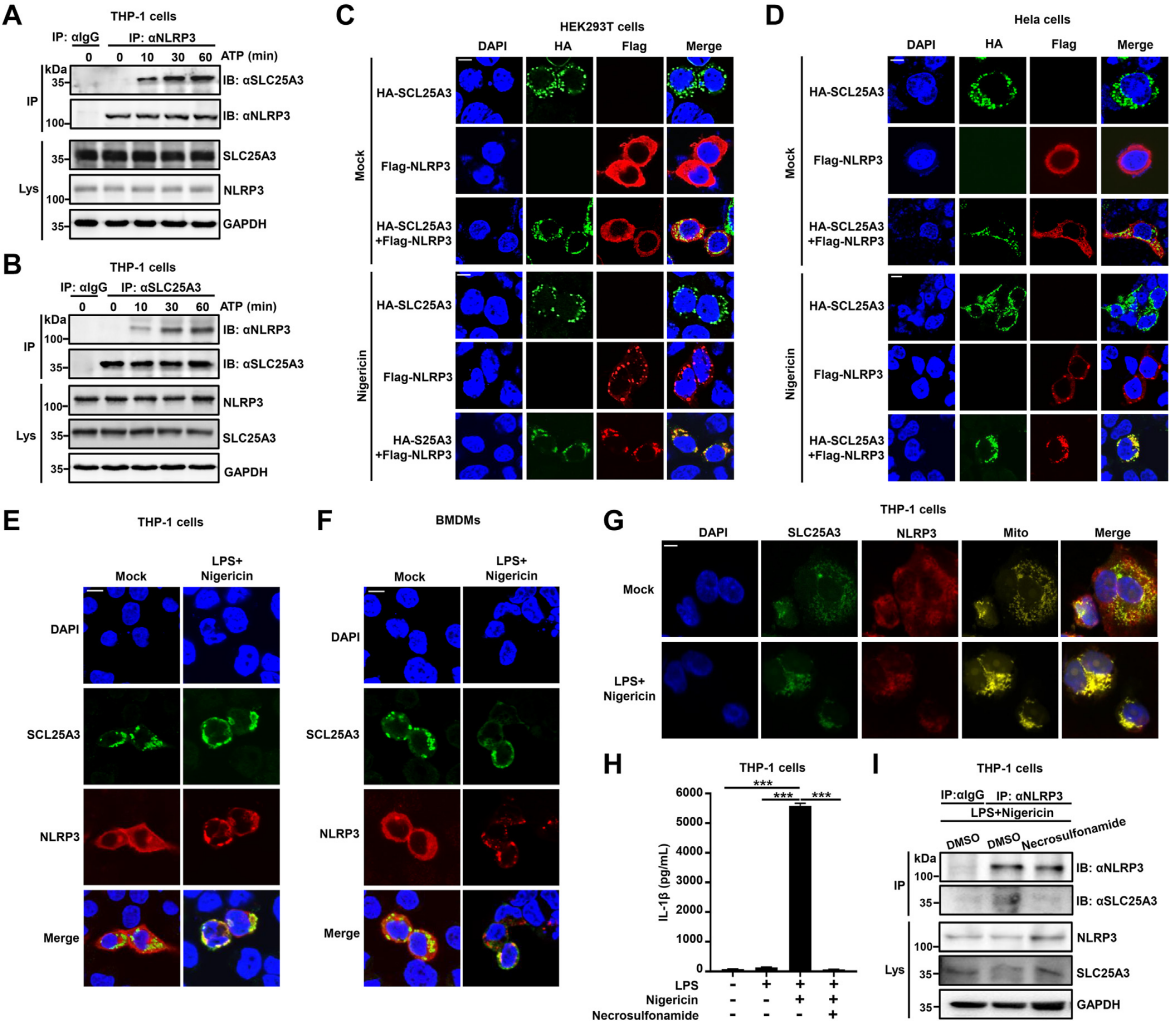
**The interaction of SLC25A3 and NLRP3 was enhanced by NLRP3 inflammasome inducers in the mitochondria.***A*,THP-1 derived macrophages were primed with LPS (100 ng/ml) for 3 h, then stimulated by ATP (5 mM) at different time points (0 min, 10 min, 30 min, or 60 min). The cell lysates were immunoprecipitated with IgG or anti-NLRP3 antibody and then immunoblotted with indicated antibodies. *B*, THP-1 derived macrophages were primed with LPS (100 ng/ml) for 3 h, then stimulated by ATP (5 mM) at different time points (0 min, 10 min, 30 min, or 60 min). The cell lysates were immunoprecipitated with IgG or anti-SLC25A3 antibody and then immunoblotted with indicated antibodies. *C*, HEK293T cells were stimulated with Mock (DMSO) or nigericin (10 μM, 1 h). The cells were immunostained with anti-HA and anti-Flag antibodies. The subcellular localizations of HA-SLC25A3 (*green*), Flag-NLRP3 (*red*), and nucleus marker DAPI (*blue*) were analyzed under confocal microscopy. The scale bar represents 10 μm. *D*, HeLa cells were stimulated with Mock (DMSO) or nigericin (10 μM, 1 h). The cells were immunostained with anti-HA and anti-Flag antibodies. The subcellular localizations of HA-SLC25A3 (*green*), Flag-NLRP3 (*red*), and nucleus marker DAPI (*blue*) were analyzed under confocal microscopy. The scale bar represents 10 μm. *E*, THP-1-derived macrophages were stimulated with Mock or LPS (100 ng/ml, 3 h) plus nigericin (10 μM, 1 h). The cells were immunostained with anti-SLC25A3 and anti-NLRP3 antibodies. The subcellular localizations of SLC25A3 (*green*), NLRP3 (*red*), and nucleus marker DAPI (*blue*) were analyzed under confocal microscopy. The scale bar represents 20 μm. *F*, BMDMs were stimulated with Mock or LPS (100 ng/ml, 3 h) plus nigericin (10 μM, 1 h), The cells were immunostained with anti-SLC25A3 and anti-NLRP3 antibodies. The subcellular localizations of SLC25A3 (*green*), NLRP3 (*red*), and nucleus marker DAPI (*blue*) were analyzed under confocal microscopy. The scale bar represents 20 μm. *G*, THP-1 derived macrophages were stimulated with Mock or LPS (100 ng/ml, 3 h) plus nigericin (10 μM, 30 min), the cells were immunostained with anti-SLC25A3 and anti-NLRP3 antibodies. The subcellular localizations of SLC25A3 (*green*), NLRP3 (*red*), mitochondrion (*yellow*) and nucleus marker DAPI (*blue*) were analyzed under confocal microscopy. The scale bar represents 20 μm. *H*, THP-1 derived macrophages treated with NSA (10 μM, 6 h) were stimulated with Mock or LPS (100 ng/ml, 3 h) plus nigericin (10 μM, 1 h), secreted IL-1β in the supernatants were analyzed by ELISA. *I*, THP-1 derived macrophages treated with NSA (10 μM, 6 h) were stimulated with Mock or LPS (100 ng/ml, 3 h) plus nigericin (10 μM, 1 h), the cell lysates were immunoprecipitated with IgG or anti-NLRP3 antibody and then immunoblotted with indicated antibodies. BMDM, bone marrow-derived macrophage; DAPI, 4′,6-diamidino-2-phenylindole; DMSO, dimethylsulfoxide; IgG, immunoglobulin G; LPS, lipopolysaccharide; SLC25A3, solute carrier family 25 member 3; NLRP3, NACHT, leucine-rich repeat, and pyrin domain-containing protein 3; NSA, Necrosulfonamide.

**Supporting Figure S2:**
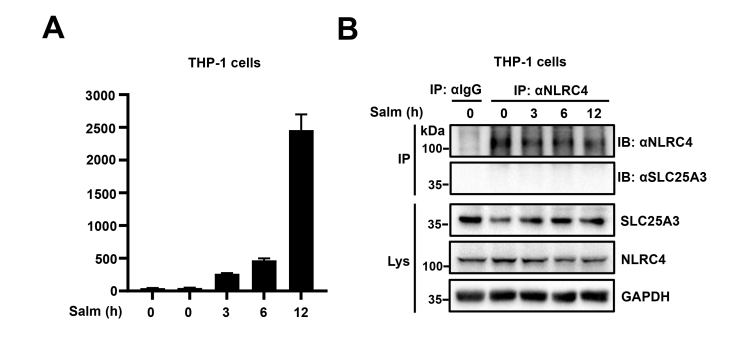


**Supporting Figure S3:**
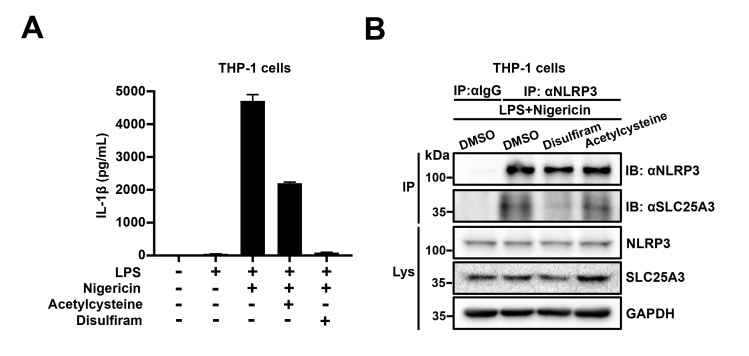


SLC25A3 could negatively regulate NLRP3 inflammasome activation. However, the underlying mechanism by which SLC25A3 regulates NLRP3 is unclear. We explore the dynamic change of interaction between SLC25A3 and NLRP3 by Co-IP and immunofluorescence. In THP-1 differentiated macrophages, the endogenous interaction between SLC25A3 and NLRP3 was enhanced by ATP (Fig. 6, *A* and *B*). We also explore the dynamic change of interaction between SLC25A3 and NLRC4 by Co-IP. *Salmonella* could activate NLRC4 inflammasome by measuring the content of secreted IL-1β (Fig. S2*A*). However, endogenous NLRC4 could not interact with SLC25A3 in the THP-1 derived macrophages stimulated by *Salmonella* at different time points (Fig. S2*B*). In HEK293T cells, exogenous colocalization between SLC25A3 and NLRP3 was increased by nigericin (Fig. 6*C*). In HeLa cells, exogenous colocalization between SLC25A3 and NLRP3 was also increased by nigericin (Fig. 6*D*). In THP-1 cells and BMDMs, the endogenous colocalization between SLC25A3 and NLRP3 was strengthened by nigericin (Fig. 6, *E* and *F*). Whether the interaction of NLRP3 and SLC25A3 can be localized on the mitochondria remains uncertain, as SLC25A3 is a mitochondrial membrane phosphate channel. The results indicated that the interaction of NLRP3 and SLC25A3 could be localized in the mitochondria in the THP-1-derived macrophages stimulated by LPS plus ATP (Fig. 6*G*). Disulfiram could directly target GSDMD and block its pore-forming activity in the cells (34). We found that disulfiram could markedly suppress secreted IL-1β induced by LPS plus nigericin, and also significantly inhibit the interaction of NLRP3 and SLC25A3 induced by LPS plus nigericin (Fig. S3, *A* and *B*). Compared to disulfiram, acetylcysteine (an inhibitor of reactive oxygen species) does not have such a significant effect (Fig. S3, *A* and *B*). Necrosulfonamide (NSA) also directly binds to GSDMD and inhibit N-terminal GSDMD oligomerization; it could also block pyroptotic cell death and IL-1β release in human monocytes/macrophages (35). We also indicated that NSA could markedly suppress secreted IL-1β induced by LPS plus nigericin, and significantly inhibit the interaction of NLRP3 and SLC25A3 induced by LPS plus nigericin in the THP-1 derived macrophages. Overall, we found that the interaction between SLC25A3 and NLRP3 in the mitochondria was strengthened by NLRP3 inflammasome inducers.

### SLC25A3 inhibits the interaction of NLRP3-NEK7 and promotes ubiquitination of NLRP3

**Figure 7:**
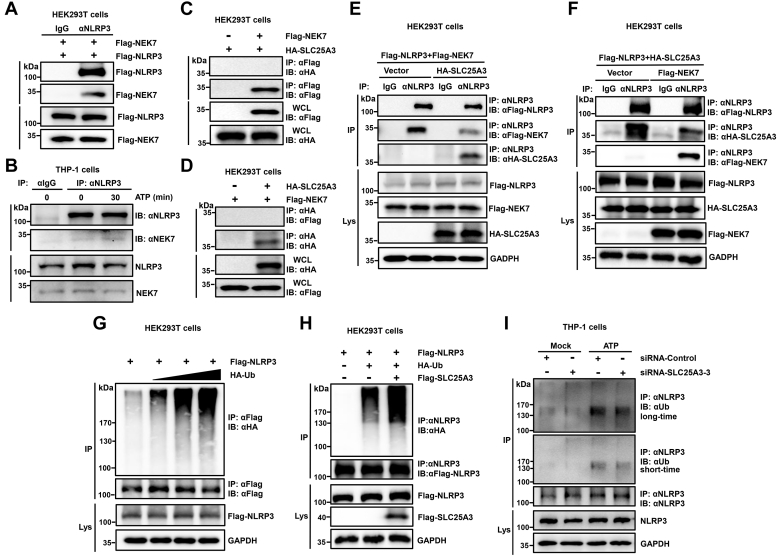
**SLC25A3 inhibits the interaction of NLRP3-NEK7 and promotes ubiquitination of NLRP3.***A*, HEK293T cells were transfected with Flag-NLRP3 and Flag-NEK7. The cell lysates were immunoprecipitated with IgG (Rabbit) or anti-NLRP3 antibodies and then immunoblotted with indicated antibodies. *B*, THP-1 derived macrophages were primed with LPS (100 ng/ml) for 6 h, then stimulated by ATP (5 mM) at different time points (0 min or 30 min). The cell lysates were immunoprecipitated with IgG or anti-NLRP3 antibody and then immunoblotted with indicated antibodies. *C*, HEK293T cells were transfected with Vector-Flag and HA-SLC25A3, or Flag-NEK7 and HA-SLC25A3. The cell lysates were immunoprecipitated with anti-Flag antibody and then immunoblotted with indicated antibodies. *D*, HEK293T cells were transfected with Flag-NEK7 and Vector-HA, or HA-SLC25A3 and Flag-NEK7. The cell lysates were immunoprecipitated with anti-HA antibodies and then immunoblotted with indicated antibodies. *E*, Flag-NLRP3 and Flag-NEK7 were co-transfected with Vector-HA or HA-SLC25A3 into the HEK293T cells as indicated in the figure. The cell lysates were immunoprecipitated with IgG (Rabbit) or anti-NLRP3 antibodies and then immunoblotted with indicated antibodies. *F*, Flag-NLRP3 and HA-SLC25A3 were cotransfected with Vector-Flag or Flag-NEK7 into the HEK293T cells as indicated in the figure. The cell lysates were immunoprecipitated with IgG (Rabbit) or anti-NLRP3 antibodies and then immunoblotted with indicated antibodies. *G*, HEK293T cells were transfected with Flag-NLRP3 and HA-Ub. The cell lysates were immunoprecipitated with anti-Flag antibodies and finally immunoblotted with indicated antibodies. *H*, HEK293T cells were transfected with Flag-NLRP3, HA-Ub, or Flag-SLC25A3 as indicated in the figure. The cell lysates were immunoprecipitated with anti-NLRP3 antibodies and finally immunoblotted with indicated antibodies. *I*, THP-1 derived macrophages were transfected with two siRNAs (siRNA-NC and siRNA-SLC25A3-3) and primed with LPS (100 ng/ml) for 6 h. THP-1 derived macrophages then were stimulated with ATP (5 mM, 30 min). The cell lysates were immunoprecipitated with anti-NLRP3 antibodies and finally immunoblotted with indicated antibodies. LPS, lipopolysaccharide; NEK7, NIMA-related kinase-7; NLRP3, NACHT, leucine-rich repeat, and pyrin domain-containing protein 3; SLC25A3, solute carrier family 25 member 3.

How SLC25A3 could negatively regulate NLRP3 inflammasome activation, we explored the deep mechanism by which SLC25A3 regulated the function of NLRP3. Firstly, we identified the interaction between NLRP3 and NEK7. The results indicated that NLRP3 could interact with NEK7 in HEK293T cells and the interaction between NLRP3 and NEK7 could be strengthened by the inducer of NLRP3 inflammasome (ATP) (Fig. 7, *A* and *B*). We also found that SLC25A3 could not interact with NEK7 in the HEK293T cells (Fig. 7, *C* and *D*). SLC25A3 or NEK7 could competitively bind to NLRP3 and suppress the interaction between NLRP3 and NEK7 or the interaction between NLRP3 and SLC25A3 (Fig. 7, *E* and *F*). We also investigated whether the interaction between SLC25A3 and NLRP3 affected the posttranslational modification of NLRP3. In the HEK293T cells, NLRP3 could be ubiquitinated in cells (Fig. 7*G*). Overexpression of SLC25A3 could promote ubiquitination of NLRP3 in the HEK293T cells (Fig. 7*H*). To study the stability of NLRP3 protein, we performed a protein decay assay with cycloheximide which blocked cellular protein synthesis. The results showed that overexpression of SLC25A3 had no effect on the stability of NLRP3 protein (Fig. S4). Knockdown of SLC25A3 could suppress ubiquitination of endogenous NLRP3 in THP-1-differentiated macrophages (Fig. 7*I*). Ubiquitination of NLRP3 always suppressed NLRP3 inflammasome activation (21, 22, 23, 24, 25, 26). That SLC25A3 promoted ubiquitination of NLRP3 and may suppress the activation of NLRP3 inflammasome. These results indicated that SLC25C3 might suppress the activation of NLRP3 inflammasome by disrupting the interaction of NLRP3-NEK7 and promoting ubiquitination of NLRP3.

## Discussion

NLRP3 inflammasome is stimulated by pathogen-associated molecular patterns and damage-associated molecular patterns to induce inflammatory responses (36, 37). Accurate and tight regulation of NLRP3 inflammasome activation is essential for host cells. It is critical to explore these mechanisms underlying repression of NLRP3 inflammasome activation. We initially showed that SLC25A3 could interact with NLRP3. Subsequently, overexpression of SLC25A3 and knockdown of SLC25A3 could regulate NLRP3 inflammasome activation in macrophages, and the interaction of NLRP3 and SLC25A3 significantly boosted in the mitochondria when NLRP3 inflammasome is activated. Detailed investigation demonstrated that the interaction between NLRP3 and SLC25A3 disrupted the interaction of NLRP3-NEK7, promoted ubiquitination of NLRP3, and finally negatively regulated NLRP3 inflammasome activation.

SLC25A3 is an inner mitochondrial membrane phosphate transporter; our data also indicated that SLC25A3 was located in the mitochondria. SLC25A3 virtually could not interact with NLRP3 without the stimulators of NLRP3 inflammasome in THP-1-derived macrophages. Perhaps, the wide distribution of the endogenous NLRP3 in the cytoplasm could explain this result. Certainly, we found that SLC25A3 could interact with NLRP3 in the HEK293T cells transfected with SLC25A3 and NLRP3 plasmids. This indicated that SLC25A3 could structurally interact with NLRP3. Interestingly, our data indicated that the interaction of NLRP3 and SLC25A3 was significantly enhanced in the mitochondria in the THP-1-derived macrophages stimulated by the stimulator of NLRP3 inflammasome. NLRP3 could be localized to the mitochondria when NLRP3 inflammasome was activated (38, 39). Our data also indicated that NLRP3 could be localized to the mitochondria in the THP-1-derived macrophages stimulated by ATP. However, NLRP3 could not interact with SLC25A3 in the space, because SLC25A3 was localized in the mitochondrial inner membrane. It seems unable to explain the interaction of SLC25A3 and NLRP3 in the THP-1-derived macrophages stimulated by ATP. Recently, it has been reported that the mitochondria was damaged early in GSDMD-mediated pyroptosis, and the N-terminal pore-forming GSDMD fragment (GSDMD-NT) caused mitochondrial damage by permeating mitochondrial inner and outer membranes to accelerate and enhance pyroptosis (40, 41). The viewpoints seem to explain that the interaction of NLRP3 and SLC25A3 could obviously be enhanced in the THP-1 derived macrophages stimulated by ATP. Damaged mitochondria may provide space for the interaction of NLRP3 and SLC25A3. Exposed SLC25A3 may suppress NLRP3 inflammasome activation by binding unassembled NLRP3. Disulfiram (an inhibitor of GSDMD, inhibits GSDMD pore formation in liposomes) could significantly suppress IL-1β secretion and the interaction of SLC25A3 and NLRP3 in the THP-1 derived macrophages. Acetylcysteine (an inhibitor of reactive oxygen species) treatment had no obvious inhibitory effect on the interaction of SLC25A3-NLRP3 compared to Disulfiram. However, Disulfiram could also block inflammatory TLR4 signaling by targeting MD-2 and its inhibition of TLR4 signaling is independent of GSDMD and caspase-1 (42). To further highlight the role of the inhibitors of GSDMD, we also used NSA rather than Disulfiram as NSA does not inhibit other innate immune pathways such as toll-like receptor signaling and GSDME-mediated cell death (35). The result also indicated NSA could significantly suppress IL-1β secretion and the interaction of SLC25A3 and NLRP3 in the THP-1 derived macrophages. These results support the above hypothesis that NLRP3 could interact with SLC25A3 in the mitochondria by GSDMD. Of course, a large number of experiments will be performed to support our hypothesis.

It has been commonly reported that interaction between targeted protein and NLRP3 negatively regulated NLRP3 inflammasome activation (21, 24, 29, 43, 44, 45). How does interaction between targeted protein and NLRP3 negatively regulate activation of NLRP3 inflammasome? Some of these described mechanisms explained why SLC25A3 suppresses NLRP3 inflammasome activation, such as ubiquitination of NLRP3 and the interaction of NLRP3-NEK7. Ubiquitination of NLRP3 is an important modification for the regulation of NLRP3 inflammasome activation (22, 23, 24, 25, 27, 29). It has been reported that E3 ubiquitin ligases, ARIH2 and TRIM65, promotes lys48-and lys63-linked ubiquitination of NLRP3, therefore suppressing NLRP3 inflammasome activation (22, 29). Recently, the report demonstrated that Maresin1 negatively regulates NLRP3 inflammasome activation by promoting K63-linked ubiquitination of NLRP3 in macrophages (25). NEK7 is an essential protein to mediate NLRP3 inflammasome assembly and activation (46, 47, 48). It has been reported that artemisinin inhibited NLRP3 inflammasome activation by suppressing interaction of NEK7 and NLRP3 (49). Licochalcone B specifically disrupted the interaction of NLRP3-NEK7 to inhibit NLRP3 inflammasome activation (19). Certainly, interaction between NLRP3 and SLC25A3 mayaffect the function of SLC25A3. The interaction possibly decreases ATP production in mitochondria, therefore suppressing NLRP3 inflammasome activation. Because NLRP3 oligomerization is dependent of ATP in the process of NLRP3 inflammasome activation (50).

In a word, we demonstrate that SLC25A3 could interact with NLRP3 and negatively regulate NLRP3 inflammasome activation. Furthermore, SLC25A3 may provide a new idea for therapeutic strategy of NLRP3 inflammasome related inflammatory diseases.

## Experimental procedures

### Cells

L929, HEK293T, and THP-1 cells were purchased from China Center of Type Culture Collection (CCTCC). L929 and HEK293T cells were cultured in Dulbecco's modified Eagle's medium purchased from Gibco supplemented with 10% fetal bovine serum (FBS), 100 U/ml penicillin, and 100 μg/ml streptomycin sulfate. THP-1 cells were cultured in RPMI 1640 purchased from Gibco supplemented with 10% FBS, 100 U/ml penicillin, and 100 μg/ml streptomycin sulfate. THP-1 was induced by phorbol-12-myristate-13-acetate (40 ng/ml) for 12 to 16 h to form THP-1 derived macrophages. BMDMs were isolated from bone marrow of 6 to 8-week-old female mice; these experiments were performed as described previously (24). Briefly, bone marrow was flushed with RPMI 1640 and collected cells were resuspended and passed through a 200-pore sized mesh. The cells were pretreated with Red Blood Cell Lysis Buffer for 5 min, and then the cells were cultured in Dulbecco's modified Eagle's medium complemented with 10% FBS, 10% to 20% L929 cells conditioned medium, 100 μg/ml streptomycin sulfate, and 100 U/ml penicillin for 5 to 6 days.

### Reagents and antibodies

Puromycin (ant-pr-1), nigericin (tlrl-nig), MDP (tlrl-mdp), poly(dA:dT)/LyoVec (tlrl-patc), Val-boroPro (tlrl-vbp-10), MSU (tlrl-msu), and Alum (tlrl-alk) were purchased from InvivoGen Biotech Co, Ltd *Salmonella* typhimurium was reserved in our laboratory. Protease Inhibitor Cocktail tablets were purchased from Roche. Lipo2000 (11668019) and TRIzol were obtained from Invitrogen. Polybrene (TR-1003-G), phorbol-12-myristate-13-acetate (P8139), ATP (A7699) and LPS (L2630) were obtained from Sigma-Aldrich. Human IL-1β ELISA Kit II (557966) and huma IL-1β ELISA Set II (557953) were purchased from BD Biosciences. Mouse IL-1β Valukine ELISA Kit (Cat#VAL601) was purchased from Novus Biologicals. NSA (HY-100573), Disulfiram (HY-B0240), and acetylcysteine (HY-B0215) were purchased from MedChemExpress Technology (MCE). LDH Cytotoxicity Assay Kit (C0017), Mito-Tracker Red CMXRos was purchased from Beyotime Technology.

GAPDH (Cat# 60004-1-lg) antibody was obtained from Proteintech. NLRP3 (D4D8T), NLRC4 (D5Y8E), IL-1β (D3U3E), IL-1β (3A6), caspase-1 (D7F10), and caspase-1 (E2Z1C) antibodies were obtained from Cell Signaling Technology. ASC (F-9) and NEK7(B-5) antibodies were purchased from Santa Cruz Biotechnology. NLRP3 (Cryo-2) antibody was purchased from AdipoGen. Anti-Flag (Cat# F3165) and Anti-hemagglutinin (HA) (Cat# H6908) antibodies were purchased from Sigma-Aldrich. Anti-Rabbit immunoglobulin G (IgG) FITC (Cat# A22120) and Anti-Mouse IgG Dylight 649 (Cat# A23610) antibodies were purchased from Abbkine. Anti-SLC25A3(ab89117) Antibody was purchased from Abcam. NLRC4 (A7382), NLRP3 (A5652), GSDMD (A20728), and Cleaved GSDMD (N Terminal) (A22523) antibodies were purchased from ABclonal.

### Real-time PCR

Total RNA extraction was performed following manufacturer’s instructions using the indicated reagents. Reaction premix was dependent with SYBR RT-PCR kits. Briefly, mixture includes 10 μl 2xSYBR Green mix, 1 μl cDNA template, 1 μl specific primers (forward (F) and reverse (R) primer each), and 8 μl ddH_2_O.

All RT-PCR primers were designed in website (https://www.ncbi.nlm.nih.gov/). The following primers were used in this study:

Human-SLC25A3-F: 5′-TGGTGTTCGTGGTTTGGCTAA-3′;

Human-SLC25A3-R: 5′-GATGTGCGCCAGAGATAAGTATT-3′;

Mouse-SLC25A3-F: 5′-GGCTCCATGAAGTATTATGCACT-3′;

Mouse-SLC25A3-R: 5′-AAACCACGAACGCCATCTTCT-3′;

Human GAPDH-F: 5′-AAGGCTGTGGGCAAGG-3′;

Human GAPDH-R: 5′-TGGAGGAGTGGGTGTCG-3′;

Mouse GAPDH-F: 5′-TTCACCACCATGGAGAAGGC-3′;

Mouse GAPDH-R: 5′-GGCATCGACTGTGGTCATGA-3′.

### Lentiviral production

pLenti-CMV vector was derived from pLenti-CMV-EGFP vector that was reserved in our laboratory. 3Flag-SLC25A3 (human) was cloned into pLenti-CMV vector. ShRNA of SLC25A3 (human) or SLC25A3 (mouse) were cloned into pLKO.1 vector. pLenti-3Flag-SLC25A3/pLKO.1-shRNA-SLC25A3, pMD2.G, and psPAX2 plasmids were cotransfected into HEK293T cells to generate lentivirus. Briefly, the culture supernatants of HEK293T cells were harvested at 36 h and 48 h after transfection. The culture supernatants were filtered through a 0.45 μm filter. The cells were infected with lentivirus plus 8 μg/ml polybrene for 24 to 36 h. Subsequently, 1 to 1.5 μg/ml puromycin was added into the culture supernatants for selection of the cells stably expressing SLC25A3 or shRNA-SLC25A3. After 5 to 7 days, the cells stably expressing SLC25A3 or shRNA-SLC25A3 were identified by qPCR and immunoblot analysis. The specific experimental protocols needs to refer to the website (http://www.addgene.org/protocols/plko/). The specific sequences of shRNAs are as follows:

Human-sh-SLC25A3-1: GCCTGCTTTGAACGTACTGTT.

Human-sh-SLC25A3-2: GTGAAGGTCTACTTCAGACTT.

Human-sh-SLC25A3-3: CAAGGGCATATTTAACGGATT.

Mouse-sh-SLC25A3-1: GCTGCTAAAGTTCGAATTCAA.

Mouse-sh-SLC25A3-2: CGACTCTGTGAAGGTCTACTT.

Mouse-sh-SLC25A3-3: GCAACATACTTGGTGAGGAAA.

### Plasmids and small interfering RNAs

Human or mouse SLC25A3 genes were cloned into pcDNA3.1(+)-3xFlag, pCAGGS-HA or pLenti-3xFlag Vectors. Human or mouse shRNA of SLC25A3 were constructed into pLKO.1 vector. That NLRP3 inflammasome related fragments or genes were constructed into pcDNA3.1(+)-3xFlag or pCAGGS-HA vectors were conserved in our laboratory. All recombinant plasmids were confirmed by DNA sequencing.

Small interfering RNAs (siRNA) specific to SLC25A3 and its negative control (siRNA-NC) were obtained from RiBo Biotech. Target sequences are listed as followed:

siRNA-NC: 5′-TTCTCCGAACGTGTCACGT-3′;

siRNA-SLC25A3-1: 5′-AGTACAAGGGCATATTTAA;

siRNA-SLC25A3-2: CTCTGGCGCACATCACTAT;

siRNA-SLC25A3-3: GACTCCGTGAAGGTCTACT.

### Western blot analysis

Firstly, cells were lysed in lyses buffer for Western blot analysis. Cell lysates were separated by 7.5 to 12.5% SDS-PAGE and then transferred onto a nitrocellulose membrane. The membranes were sealed in PBS with 0.1% Tween 20 containing 5% nonfat dried milk for 45 min at room temperature (RT) and then were incubation with first antibodies at 4 °C overnight. Next, the membranes were incubated with second antibodies for 45 min at RT. Finally, the membranes were detected with the Western enhanced chemiluminescence substrate (Bio-Rad).

### ASC oligomerization assay

The experiment was performed as described in detail previously (51). Briefly, THP-1 derived macrophages were lysed in buffer (50 mM Tris, pH 7.5, 150 mM NaCl, 1% NP-40, 5 mM EDTA, and 10% glycerol). The pellets of the lysates were washed with PBS and then cross-linked using disuccinimidyl suberate. Finally, the pellets were mixed with 2 × SDS loading buffer for ASC oligomerization analysis.

### LDH cytotoxicity assay

LDH cytotoxicity assay kit was used to determine cytotoxicity by measuring LDH activity in damaged cells. LDH was a stable cytoplasmic enzyme present in all cells. Once cells are impaired by chemicals, stress, injuries, or intercellular signals, LDH is rapidly released into the culture medium. The supernatants from the culture of experimental cells were examined by LDH cytotoxicity assay kit according to manufacturer’s instructions.

### Coimmunoprecipitation

Firstly, the cells were washed with precold PBS and lysed in RIPA lyses buffer (50 mM Tris–HCl (pH 7.4), 150 mM NaCl, 1% (vol/vol) NP-40, 1 mM EDTA, and 5% (vol/vol) glycerol) containing protease inhibitors cocktails for 10 to 20 min; secondly, the lysates were centrifuged for 10 to 15 min at 4 °C, a part of the lysates were saved as input, the rest of the lysates were incubated with the indicated antibodies at 4 °C overnight for immunoprecipitation; thirdly, the lysates were incubated with protein G-agarose for 2 h, then the beads were washed for 5 to 6 times by RIPA washing buffer (50 mM Tris–HCl (pH7.4), 300 mM NaCl, 1% (vol/vol) NP-40, 1 mM EDTA, and 5% (vol/vol) glycerol); finally, the beads were reconstituted in 50 μl × SDS loading buffer.

### Immunofluorescence microscopy

Firstly, the cells were washed three times with precold PBS, fixed with 4% paraformaldehyde for 15 min, and then the cells were permeabilized with PBS containing 0.5% Triton X-100 for 5 min, and blocked with PBS containing 5% bovine serum albumin at RT for 45 min; secondly, the cells were incubated with the indicated antibody at 4 °C overnight, and followed by incubation with fluorescent secondary antibodies; thirdly, the cells were incubated with 4′,6-diamidino-2-phenylindole for 5 min in 37 °C after washing three times; finally, the cells were analyzed using a confocal laser scanning microscope.

### Statistical analyses

All experiments were reproducible, and each set was repeated at least three times. For data with a normal distribution and homogeneity of variance, differences between the two groups were statistically analyzed by a two-tailed Student *t* test. Statistical significance was valued based on the *p* value. ∗indicates *p* <0.05; ∗∗ indicates *p* <0.01; ∗∗∗ indicates *p* <0.001. A *p* <0.05 was considered statistically significant.

## Data availability

All data are available within the article.

## Supporting information

This article contains supporting information.

## Conflict of interest

The authors declare that they have no conflicts of interest with the contents of this article.

## Figures and Tables

**Supporting Figure S4:**