# Supplementary material for: SLC25A3 negatively regulates NLRP3 inflammasome activation by restricting the function of NLRP3
Source: J Biol Chem. 2024 Mar 27;300(5):107233. doi: 10.1016/j.jbc.2024.107233 (PMC11067542; doi:10.1016/j.jbc.2024.107233)
Supplement: Supporting Figures S1–S4 legends [file mmc1.docx]

**Supporting information**

**Figure S1. (A-C)** HEK293T cells were transfected with Vector, Vector-Caspase-1 and Vector-IL-1β, Vector-AIM2/NLRC4/NLRP1 and Vector-ASC, or Vector-AIM2/NLRC4/NLRP1, Vector-ASC, Vector-Caspase-1 and Vector-IL-1β as indicated in the figure. Secreted IL-1β in the supernatants were analyzed by ELISA. **(D-E)** HEK293T cells were co-transfected with Vector-AIM2/NLRC4/NLRP1, Vector-ASC, Vector-Caspase-1 and Vector-ASC four plasmids, along with a series of Vector-HA-SLC25A3. Secreted IL-1β in the supernatants were analyzed by ELISA (top), and the cell lysates were immunoprecipitated with with indicated antibodies (bottom).

**Figure S2**. (A)THP-1 macrophages were stimulated by Salmonella Typhimurium (MOI=2) at different timepoints (0h, 3h, 6h or 12h). Secreted IL-1β in the supernatants were analyzed by ELISA. (B) THP-1 macrophages were primed with LPS (100 ng/ml) for 3h, then stimulated by Salmonella (MOI=2) at different timepoints (0h, 3h, 6h or 12h). The cell lysates were immunoprecipitated with IgG or anti-NLRC4 antibody and then immunoblotted with indicated antibodies.

**Figure S3.** (A)THP-1 derived macrophages treated with Acetylcysteine (10mM, 6h) or Disulfiram (10μM, 2h) were stimulated with Mock or LPS (100 ng/ml, 3h) plus nigericin (10μM, 1 h), secreted IL-1β in the supernatants were analyzed by ELISA. (B)THP-1 derived macrophages treated with Acetylcysteine (10mM, 6h) or Disulfiram (10μM, 2h) were stimulated with Mock or LPS (100 ng/ml, 3h) plus nigericin (10μM, 1 h), the cell lysates were immunoprecipitated with IgG or anti-NLRP3 antibody and then immunoblotted with indicated antibodies.

**Figure S4**. HEK293T cells were co-transfected with Vector-Flag-NLRP3 and Vector-HA, or Vector-Flag-NLRP3 and Vector-HA-SLC25A3, then were treated with CHX (50 mg/ml) in different timepoints (0h, 1h, 2h, 4h, 8h or 12h); the cell lysates were immunoblotted with indicated antibodies.
